# Comparison of Oropharyngeal Dysphagia in Brazilian Children with Prenatal Exposure to Zika Virus, With and Without Microcephaly

**DOI:** 10.1007/s00455-020-10173-4

**Published:** 2020-09-04

**Authors:** Danielle Maria da Silva Oliveira, Demócrito de Barros Miranda-Filho, Ricardo Arraes de Alencar Ximenes, Ulisses Ramos Montarroyos, Celina Maria Turchi Martelli, Elizabeth B. Brickley, Mariana de Carvalho Leal Gouveia, Regina Coeli Ramos, Maria Ângela Wanderley Rocha, Thalia Velho Barreto de Araujo, Sophie Helena Eickmann, Laura Cunha Rodrigues, Jeyse Polliane de Oliveira Soares Bernardes, Maria Helena Teixeira Pinto, Karina Polo Norte Danda Soares, Claudia Marina Tavares de Araújo, Maria de Fátima Pessoa Militão-Albuquerque, Ana Célia Oliveira dos Santos

**Affiliations:** 1 Faculdade de Ciência Médicas/Hospital Universitário Oswaldo Cruz/ Universidade de Pernambuco, Setor NIR- ZIKA, Rua Arnóbio Marques, no. 310, Santo Amaro, Recife, PE CEP 50100-130 Brazil; 2 Universidade Federal de Pernambuco, Av. Prof. Moraes Rego, 1235 - Cidade Universitária, Recife, Pernambuco Brazil; 3 Instituto Aggeu Magalhães, Campus da UFPE - Av. Prof. Moraes Rego, S/N - Cidade Universitária, Recife, Pernambuco Brazil; 4 London School of Hygiene and Tropical Medicine, London, Keppel St, Bloomsbury, London, WC1E 7HT UK

**Keywords:** Oropharyngeal dysphagia, Zika virus, Zika-related microcephaly, Zika-exposed children without microcephaly, Congenital Zika syndrome

## Abstract

Severe brain damage associated with Zika-related microcephaly (ZRM) have been reported to result in oropharyngeal dysphagia (OPD); however, it is unknown if OPD presents in children with prenatal Zika virus (ZIKV) exposure but only mild or undetectable abnormalities. The aims of this study were: to compare the frequency and characteristics of OPD in children with ZRM and in children without microcephaly born to mothers who tested polymerase chain reaction positive (PCR+) for ZIKV during pregnancy; and to investigate the concordance of caregiver reports of OPD with the diagnosis from the clinical swallowing assessment (CSA). Between Mar/2017 and May/2018, we evaluated 116 children (*n* = 58 with microcephaly, *n* = 58 children without microcephaly born to ZIKV PCR + mothers) participating in the Microcephaly Epidemic Research Group (MERG) cohort of children born during the 2015–2016 ZIKV epidemic in Pernambuco, Brazil. To assess OPD we used: a CSA; a clinical assessment of the stomatognathic system; and a questionnaire administered to caregivers. The frequency of OPD was markedly higher in children with ZRM (79.3%) than in the exposed but normocephalic group (8.6%). The children with microcephaly also presented more frequently with anatomic and functional abnormalities in the stomatognathic system. There was a high degree of agreement between the caregiver reports of OPD and the CSA (κ = 0.92). In conclusion, our findings confirm that OPD is a feature of Congenital Zika Syndrome that primarily occurs in children with microcephaly and provide support for policies in which children are referred for rehabilitation with an OPD diagnosis based on caregiver report.

## Introduction

Zika virus (ZIKV) gained global attention in 2015 with the emergence of the microcephaly epidemic in the Northeast region of Brazil [1, 2]. Children with Zika-related microcephaly (ZRM) have severe damage to the central nervous system with brain dysgenesis and intracranial calcifications [3]. Alongside microcephaly, the epidemic of Congenital Zika Syndrome (CZS) introduced an increase in the frequency of other severe neurological abnormalities. Similar to children with cerebral palsy, children congenitally infected with ZIKV have been reported to have brain damage associated with delayed motor, language, and cognitive development, visual difficulties, and dysphagia [3–5].

Dysphagia is a disorder, usually related to neurological impairment, which affects the ability to swallow and may lead to complications, such as aspiration pneumonia, dehydration, and malnutrition [6]. Dysphagia may be observed at all stages of swallowing (i.e., the oral, pharyngeal, and esophageal stages) and affects both the automatic swallowing of saliva and the voluntary swallowing of food. Different cortical regions, including the pre- and postcentral gyri and the insula [7, 8], activate the automatic and voluntary processes of swallowing, and recently, central pattern generators in the brainstem and spinal cord have been described to be responsible for the rhythmic pattern of sucking–swallowing–breathing movements [9]. ZIKV infection can lead to calcification of the cerebellum, cerebral parenchyma, basal ganglia, brainstem, and periventricular region, and also cause cerebral atrophy, ventricular dilation, and, in some cases, spinal cord atrophy that may result in varying degrees of swallowing disorders [10, 11].

Few studies have described dysphagia in CZS, particularly in the exposed population without microcephaly [12, 13]. This study has aimed to investigate the frequency and characteristics of the dysphagia in the two first stages of swallowing process, i.e. oropharyngeal dysphagia (OPD), in children with ZRM and in children born to mothers who tested PCR+ for Zika virus (ZIKV) during pregnancy, without microcephaly and independently of the presence of other symptoms, and to compare the swallowing function between the two groups through a clinical swallowing assessment (CSA). We also evaluated if the caregiver report could be used to refer the children to a specialized service, by testing the degree of agreement between the caregiver reports of OPD and the CSA diagnosis.

## Methods

### Study Design and Participants

This is a cross-sectional study comparing the frequency and characteristics of OPD in two groups of children. Between March 2017 and May 2018, investigators affiliated with the Microcephaly Epidemic Research Group (MERG) enrolled children born during the 2015–2017 ZIKV epidemic in Pernambuco, Brazil to this study. The inclusion criteria for each group were: (i) group 1—born during the microcephaly epidemic (from May 2015 to April 2017) with microcephaly diagnosed either at birth or during the first months of life; presenting with either laboratory evidence of ZIKV infection or imaging abnormalities compatible with CZS; and (ii) group 2—children without microcephaly born to women with a rash and ZIKV infection during pregnancy confirmed with reverse transcription polymerase chain reaction (RT-PCR) (based on the primers of Lanciotti et al. [14]), from now on referred as Zika-exposed children without microcephaly. For infants born at term (i.e., ≥ 37 weeks of gestation), microcephaly was defined as a head circumference (HC) that measured ≤ 2 standard deviations below the mean for both age and sex, according to the World Health Organization (WHO) 2006 growth chart [15]. For preterm infants, microcephaly was defined as a HC that measured ≤ 2 standard deviations below the mean for gestational age and sex, according to the Intergrowth 2015 growth chart [16].

### Assessing Dysphagia

Children were evaluated at the Rehabilitation Center of the Fundação Altino Ventura, by a study team trained and supervised by an experienced speech therapist from the Interdisciplinary Nucleus for the Rehabilitation of Children with CZS at the Hospital Universitário Oswaldo Cruz (NIR-ZIKA-HUOC), a referral center for children with CZS, in Recife, Pernambuco, Brazil. The evaluators received training for at least 4 weeks prior to assessing for children for OPD. Evaluations were based on: (i) a questionnaire administered to caregivers, (ii) a clinical assessment of the stomatognathic system, and (iii) a clinical swallowing assessment (CSA). The three instruments were adapted from the Protocol for the Assessment of Pediatric Dysphagia (PAD-PED), [17] which was developed by the Pontifical Catholic University of São Paulo with the purpose of conducting the clinical assessment of childhood OPD in Brazil. All data were recorded on standardized case report forms, which were reviewed by an experienced speech therapist. Assessments were repeated if inconsistencies were detected.

In the clinical assessments, team members investigated physical abnormalities in the development of the stomatognathic system and functional abnormalities in the swallowing dynamics. With regard to the development of the stomatognathic system, the following variables were described: resting posture, tone/strength and mobility of lips, tongue, and cheeks. In children with Zika-related microcephaly, tongue mobility was observed through sensory stimuli since these children did not effectively respond to verbal commands. We defined appropriate resting tongue posture when the tip of tongue is placed behind the incisive papilla.

OPD was defined and classified based on PAD-PED [17]. Swallowing in the oral and pharyngeal phases was primarily assessed using food provided by the caregivers in the consistency usually consumed by the child and offered using the child’s own utensils, baby bottle, cups, and spoons. In the absence of food, swallowing was assessed using water as a liquid consistency, water thickened with xanthan gum, and a starch-based biscuit as a solid consistency. OPD was classified as: absent (i.e., no abnormalities in swallowing), mild (i.e., the presence of at least one sign that indicated compromised oropharyngeal transit, with no signs suggestive of aspiration), moderate (i.e., compromised oropharyngeal transit, with the presence of at least one sign suggestive of aspiration, but the protective mechanisms are preserved, allowing the clearance of the lower airways), and severe (i.e., compromised oropharyngeal transit, with signs suggestive of aspiration and an absence of protective mechanisms).

In the oral phase, the following variables were assessed: loss of food from mouth (i.e., food lost from the mouth due to lack of oral control), increased oral transit time (measured with a digital timer, activated when the food was placed in the participant's oral cavity and terminated at the moment when oral transit was observed for the pharyngeal phase of swallowing (normal duration for liquids: 0.35 to 1.54 s; for pasty food: 0.39 to 1.05 s; and for solid food: 1 to 12.8 s [18]), oral stasis of food (i.e., the child does not swallow the food offered but keeps it in the oral cavity), and difficulties in the chewing process (i.e., when solid food had already been included in the child’s diet). In the pharyngeal phase, the following variables were assessed: laryngeal elevation (assessed by the placing the index finger to the hyoid bone); abnormal pharyngeal transit (when the laryngeal excursion was incomplete, i.e., the larynx was not elevated sufficiently and the clearance of food during swallowing did not occur properly, causing a wet voice and repeated swallows); signs of OPD (i.e., cough, choking, dyspnea, and cyanosis).

Signs indicative of laryngotracheal penetration/aspiration were characterized by analyzing the vocal quality of the children and the cervical auscultation using a pediatric stethoscope, which permits the auscultation of high- and low-frequency sounds with high acoustic sensitivity [19]. The children were assessed in the process of vocalization or crying through the auditory perception of the speech therapist after stimulation to vocalization or spontaneous crying. A wet vocal quality was considered as a sign of OPD. Abnormalities during cervical auscultation were defined as the presence of respiratory sounds before and after swallowing saliva and food.

During feeding, the body posture and head position of the child were observed. Body posture was considered to be appropriate when the child’s caregiver supported and positioned him/her appropriately during feeding or when the child kept the trunk upright without caregiver support. Head alignment was considered to be appropriate when the child’s head was supported at an angle of 90° to the ground during feeding, whether aided by the caregiver or not.

Due to the low numbers, moderate and severe OPD were grouped together in the analysis.

### Statistical Analyses

Chi-squared tests were used to compare the distribution of categorical variables in children with microcephaly and Zika-exposed children without microcephaly. The Fisher’s exact test was used when cells contained 5 or fewer observations. The Kappa test was used to assess the agreement between caregiver reports and the CSA results for OPD. All *p* values were from 2-sided statistical tests, and all analyses were performed using Stata, 8.0 (Stata Corp LP, College Station, Texas, USA).

### Ethics

The study was approved by the Research Ethics Committee at the Hospital Universitário Oswaldo Cruz (CAAE 52803316.8.0000.5192) and was conducted in accordance with the Declaration of Helsinki. Caregivers provided written informed consent for the children to participate in the study. Children who presented swallowing disorders were referred for complementary exams and referred to the NIR-ZIKA-HUOC or another rehabilitation unit close to their residence.

## Results

**Fig. 1:**
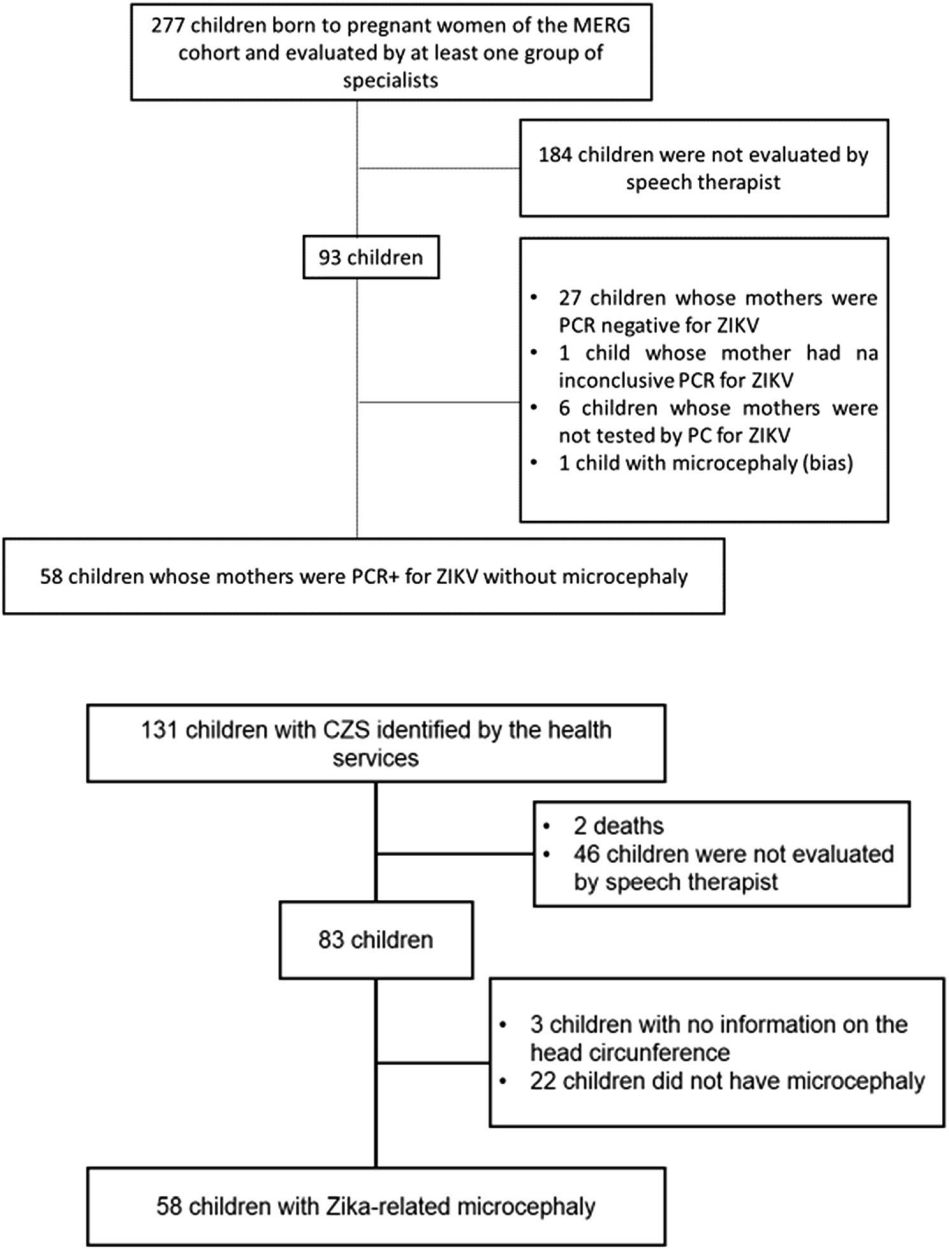
Flowchart of study sample composition

A total of 116 children, including 58 Zika-exposed children without microcephaly and 58 children with Zika-related microcephaly, were enrolled in the study (Fig. 1). Overall, 62 (53.4%) children were female. Participants’ ages ranged between 4 and 36 months, with a mean of 19.4 months, SD 6.2 (18.6 months, SD 5.0 for Zika-exposed children without microcephaly; mean of 20.3 months, SD 7.2 for the Zika-related microcephaly group). Low birth weight (below 2500 g) was more frequent in children with Zika-related microcephaly (32.7%) than in Zika-exposed children without microcephaly (12.3%) (Table 1). The mean birth weight in children with Zika-related microcephaly was 2,669.7 g (SD 571.4), significantly lower than in the group of Zika-exposed children without microcephaly (3,318.1 g, SD 586.4).

**Table 1:** Comparison of birth weight and characteristics of OPD based on data from caregiver reports on feeding and related difficulties between the groups studied—Pernambuco/2017–2018

	Zika-exposed children without microcephaly (*n* = 58)	Children with congenital Zika syndrome with microcephaly (*n* = 58)	*p* value, *χ*^2^
Birth weight			
< 2500 g	7 (12.5%)	18 (32.7%)	0.011
> 2500 g	49 (87.5%)	37 (67.3%)	
Caregiver report of OPD			
No	55 (94.8%)	34 (58.6%)	< 0.001*^1^
Yes	3 (5.2%)	24 (41.4%)	
Hospitalization during the previous 6 months			
No	48 (82.8%)	34 (58.6%)	**0.004**
Yes	10 (17.2%)	24 (41.4%)	
Alternative food route			
No	55 (94.8%)	46 (79.3%)	0.012*^1^
Yes	3 (5.2%)	12 (20.7%)	
Type of alternative food route			
Tube (nasogastric)	3 (100%)	9 (75%)	0.484*^1^
Gastrostomy		3 (25%)	
Dysphagia symptoms during feeding			
Cough			
Yes	8 (13.8%)	29 (50%)	** < 0.001**
No	50 (86.2%)	29 (50%)	
Choking			
Yes	2 (3.4%)	23 (39.7%)	< 0.001*^1^
No	56 (96.6%)	35 (60.3%)	
Cyanosis			
Yes	1 (1.7%)	4 (6.9%)	0.182*^1^
No	57 (98.3%)	50 (93.1%)	
Others			
Yes	2 (3.6%)	7 (12.3%)	0.081*^1^
No	55 (96.4%)	50 (87.7%)	

Significant *p* values are given in bold

The number of individuals varied due to missing values/*^1^—Fisher Test

Based exclusively on caregiver reports, the frequency of OPD in children with Zika-related microcephaly was approximately 13 times higher (67.2%) than in the group of Zika-exposed children without microcephaly (5.2%). The frequency of hospitalization during the previous 6 months was also higher in the group of children with microcephaly (41.4% versus 17.2%). Respiratory infection was the most frequent cause of hospitalization in both groups: 41.4% in the Zika-related microcephaly group and 17.2% in the Zika-exposed children without microcephaly group. Of the 12 (20.7%) children with Zika-related microcephaly who required an alternative route for feeding, 9 (75%) used a nasogastric tube and 3 (25%) had a gastrostomy; among the 3 (5.2%) Zika-exposed children without microcephaly who required an alternative route of feeding, all 3 (100%) used a nasogastric tube. The groups also differed in terms of the frequency of coughing and choking reported during feeding, which were approximately four times more common in the group with microcephaly. Reports of cyanosis and other complaints (i.e., nasal reflux, regurgitation, and dyspnea during feeding) did not differ between the groups.

In the physical assessments, statistically significant differences (p < 0.001 for all) were observed amongst Zika-exposed children without microcephaly and Zika-related microcephaly (Table 2). While 78.9% of the children with Zika-related microcephaly were fed by caregivers while held in an inappropriate body position, for example unstabilized trunk and head, in flexion or extension, only 14.1% of Zika-exposed children without microcephaly no had suboptimal body posture during feeding. In both groups, more than 70% of the children presented appropriate head alignment during feeding, although in the Zika-exposed children without microcephaly this percentage was even higher (*p* = 0.007). Similarly, in the assessment of the stomatognathic system, deficits in the resting posture, tonus and protrusion of the lips and tongue, indicating orofacial hypotonia, were significantly more prevalent amongst children with Zika-related microcephaly. It was also observed that significantly more children with Zika-related microcephaly presented increased cheek tone and ogival hard palate. In addition, deficits in lip and tongue retraction and differences of the lingual frenulum (e.g., short, anterior or invisible frenulum) were relatively uncommon in both groups, but occurred at a non-significantly higher frequency in children with Zika-related microcephaly. Soft palate anatomical anomaly (bifid uvula) was observed in only one child, who was in the group of Zika-exposed children without microcephaly.

**Table 2:** Comparison of stomatognathic system characteristics between the groups—Pernambuco-2017–2018

	Zika-exposed children without microcephaly (*n* = 58)	Children with congenital Zika syndrome with microcephaly (*n* = 58)	*p* value, *χ*^2^
Body position during feeding*^3^			
Inappropriate	8 (14.1%)	45 (78.9%)	** < 0.001**
Appropriate	50 (85.9%)	12 (21.1%)	
Head position during feeding*^4^			
Inappropriate	3 (5.3%)	13 (22.4%)	**0.007**
Appropriate	54 (94.7%)	45 (77.6%)	
Resting lip posture			
Occluded	45 (70.3%)	18 (31.1%)	** < 0.001**
Half open	12 (20.7%)	40 (68.9%)	
Lip tonus			
Appropriate for age	50 (86.4%)	21 (36.2%)	< **0.001**
Inappropriate	8 (13.6%)	37 (63.8%)	
Mobility of lips			
Retraction (smile)			
Yes	56 (96.6%)	50 (86.2%)	0.047*^1^
No	2 (3.4%)	8 (13.8%)	
Pursing of lips			
Yes	56 (96.6%)	26 (44.8%)	0.000*^1^
No	2 (3.4%)	32 (55.2%)	
Resting tongue posture			
Appropriate (behind the upper teeth)	26 (44.1%)	11(18.9%)	**0.003**
Inappropriate	32 (55.9%)	47 (81.1%)	
Tongue tonus			
Normal	3 (5.2%)	16 (27.6%)	< 0.001*^1^
Anomalies	55 (94.8%)	42 (72.4%)	
Tongue mobility			
Retraction			
Yes	57 (98.3%)	50 (86.2%)	0.015*^1^
No	1 (1.7%)	8 (13.8%)	
Protrusion			
Yes	58 (100%)	50 (86.2%)	< 0.015*^1^
No	–	17 (29.3%)	
Lingual frenulum			
Typical	57 (98.3%)	54 (93.1%)	0.182*^1^
Shortened	1 (1.7%)	4 (6.9%)	
Cheek tone			
Appropriate	52 (89.7%)	22 (37.9%)	** < 0.001**
Increased	1 (1.7%)	3 (5.2%)	
Decreased	5 (8.6%)	33 (56.9%)	
Hard palate			
Appropriate	55 (94.8%)	42(72.4%)	0.001*
Inappropriate	3 (5.2%)	16 (27.6%)	
Soft palate			
Appropriate	57 (98.3%)	58 (100%)	0.500*^1^
Inappropriate	1 (1.7%)	–	

Significant *p* values are given in bold

The number of individuals varied due to missing values/*^1^- Fisher Test

In the functional assessment of swallowing in the children with prenatal exposure to ZIKV, disorders in the oral and pharyngeal phases of swallowing, which are associated with OPD, occurred more frequently in children with Zika-related microcephaly than in Zika-exposed children without microcephaly. Statistically significant differences between the groups were observed in the frequency of almost all characteristics examined. Most of the Zika-exposed children without microcephaly presented with mild or moderate OPD and had greater impairment in the oral phase of swallowing, with no signs of bronchoaspiration. In this group, the frequency of OPD symptoms related to the oral swallowing phase ranged from 1.7 to 8.6%, while in children with Zika-related microcephaly this frequency was significantly higher, from 36.2 to 75.9% (Table 3). The difficulties observed that may be associated with a dysfunction of the oral phase of swallowing, such as loss of food from the mouth, abnormal cervical auscultation and oral food stasis, were more frequently observed in the children with microcephaly (ranging from 56.9 to 75.9%). The difficulties indicating dysfunction of the pharyngeal phase of deglutition, such as coughing, choking, reduction of laryngeal elevation and abnormal pharyngeal transit time, were also more frequently observed amongst children with Zika-related microcephaly (ranging from 21 to 31%), while this did not exceed 6% in Zika-exposed children without microcephaly (Table 3).

**Table 3:** Clinical swallowing assessment (CSA) Between the groups studied- Pernambuco-2017–2018

	Zika-exposed children without microcephaly (*n* = 58)	Children with congenital Zika syndrome with microcephaly (*n* = 58)	*p* value, *χ*^2^
Food escaping from mouth			
Yes	5 (8.6%)	40 (69.9%)	** < 0.001**
No	53 (91.4%)	18 (31.1%)	
Vocal quality			
Normal	57 (98.3%)	56 (96.6%)	0.494*^1^
Hoarse/wet	1 (1.7%)	2 (3.4%)	
Cervical auscultation			
Normal	56 (96.6%)	25 (43.1%)	< 0.001*^1^
Respiratory sounds present	2 (3.4%)	33 (56.9%)	
Increase in oral transit time			
Yes	4 (6.9%)	44 (75.9%)	< 0.001*^1^
Not	54 (93.1%)	14 (24.1%)	
Food stasis in the oral cavity			
Yes	2 (3.4%)	41 (70.7%)	** < 0.001**
Not	56 (96.6%)	17 (29.3%)	
Difficulties in the chewing process			
Yes	1 (1.7%)	21 (36.2%)	** < 0.001**
No	49 (84.5%)	22 (37.9%)	
No applicable	8 (13.8%)	15 (25.9%)	
Cough			
Yes	3 (5.2%)	7 (12.1%)	**0.001**
Not	55 (94.8%)	51 (89.9%)	
Choking			
Yes	2 (3.4%)	7 (12.1%)	0.078
Not	56 (96.6%)	51 (87.9%)	
Changes in the respiratory pattern			
Yes	1 (1.7%)	12 (20.7%)	< 0.001*^1^
Not	57 (94.8%)	46 (79.3%)	
Decrease laryngeal elevation			
Yes	–	18 (31%)	< 0.001*^1^
Not	58 (100%)	40 (69%)	
Increased pharyngeal transit time			
Yes	3 (5.2%)	12 (20.7%)	< 0.001*^1^
Not	55 (94.8%)	46 (79.3%)	
OPD in CSA			
Absent	53 (91.4%)	12 (20.7%)	< 0.001*^1^
Present	5 (8.6%)	46 (79.3%)	
Classification of dysphagia			
Normal swallowing	53 (91.4%)	12 (20.7%)	< 0.001*^1^
Mild dysphagia	3 (5.2%)	12 (20.7%)	
Moderate/severe dysphagia	2 (3.4%)	34 (58.4%)	

Significant *p* values are given in bold

The number of individuals varied due to missing values/*^1^- Fisher Test

Based on the CSA, the vast majority (79.3%) of children with Zika-related microcephaly were diagnosed with OPD, while in the Zika-exposed children without microcephaly the frequency of OPD was only 8.6%. In the group of children with Zika-related microcephaly, 20.7% presented with mild OPD, 32.8% with moderate OPD, and 25.8% with severe OPD. These last two categories were combined in Table 3. In the group of Zika-exposed children without microcephaly, the frequency of mild OPD was 5.2% and moderate and severe OPD was 1.72% (one case for each). When comparing a diagnosis of OPD based on caregiver reports with the CSA, the Kappa coefficient was 0.92, thereby suggesting a very high degree of concordance between the indicators.

## Discussion

OPD, as defined in the CSA, was approximately 9-times more common amongst children with Zika-related microcephaly (79.3%) than in the ZIKV-exposed children without microcephaly (8.47%), and there was an excellent degree of agreement between the identification of OPD based on caregiver report and the CSA. Physical and functional differences were observed across children in both groups. Children with Zika-related microcephaly were more likely than Zika-exposed children without microcephaly to have an inappropriate resting posture while feeding, abnormalities related to the movement and tone of the stomatognathic system, and OPD. Overall, the results suggest that OPD is a clinically significant problem in children with Zika-related microcephaly and highlight the urgent need for more research into rehabilitation and therapeutic strategies to support affected children.

The frequency of OPD in Zika-exposed children without microcephaly was higher than that observed in the general population, and for those with Zika-related microcephaly, the frequency was similar to that of children with cerebral palsy [20]. A study in the United States that reviewed the records of 569,000 children, aged 3 to 17 years, estimated a frequency of OPD of 0.9% in the general population of children [21]. In contrast, studies of children with cerebral palsy have estimated the frequency of OPD to be between 30 and 80% [20–25], similar percentages to that observed in the Zika-related microcephaly group and higher than that observed in the exposed group of children without microcephaly. The high concordance between caregiver reports and CSA regarding OPD is in line with the observations of Benfer et al. [26] in children with cerebral palsy and reinforces the value of engaging with and listening to caregivers during medical consultations.

While Zika-exposed children without microcephaly presented with feeding postures and stomatognathic systems that were more similar to the general population [27], children with Zika-related microcephaly more frequently presented with postural difficulties and anomalies in the stomatognathic system, which were similar to those found in children with cerebral palsy [28]. Although nearly three-quarters of the children with Zika-related microcephaly demonstrated inappropriate body postures during feeding [29], more than 70% of the children in both groups maintained appropriate head position. It is important to highlight that, in the wake of the ZIKV epidemic and cases of microcephaly, the Brazilian Ministry of Heath has created protocols for assessing and caring for children with microcephaly, which may have provided caregivers with prior knowledge of basic care, including feeding posture [30, 31]. Moreover, it was observed that children with microcephaly presented more changes in the tongue posture, with the tongue on the floor of the mouth, with decreased tone and mobility, rather than maintaining the tip of the tongue against the palatal papilla behind the central incisors [32, 33].

A high frequency of oral food stasis and increased oral transit, that may be attributed to central neurological dysfunction, was observed amongst children with OPD in both groups, although was much lower in the group without microcephaly. These results provide evidence that the oral phase of swallowing is substantially affected by congenital ZIKV infection and, even in the group of Zika-exposed children without microcephaly, it is far from negligible.

OPD in children with CZS may occur due to several factors usually associated with cortical injury, which can affect or control neuromotor swallowing. Between 60 and 70% of children with Zika-related microcephaly presented lip, tongue or cheek hypotonia, which may have played a role in the high frequency of OPD observed in this group, as has been previously described in relation to severe cerebral palsy of other etiologies [20]. In addition, calcifications in the cerebral cortex of children with microcephaly may also cause neurophysiological disorders related to cortical motor control of swallowing [12, 34].

The frequency of low birth weight was lower in the group of Zika-exposed children without microcephaly than in the group of children with microcephaly. When the group of Zika-exposed children without microcephaly was compared to the national data for live births, this frequency of low birth weight was modestly elevated (12.5% versus 8.5%), and we note that low birth weight has been more associated with feeding disorders that occur in early life [35, 36].

## Limitations

This investigation had strengths and limitations. To date, this is the largest published study to investigate the frequency of OPD in children with CZS and the first to examine swallowing in Zika-exposed children without microcephaly. The broad inclusion criteria enabled us to assess OPD across the full spectrum of CZS. In this investigation, we used instruments to assess OPD that have not yet been standardized and/or validated in the context of congenital ZIKV infections. To overcome this limitation, we incorporated a multimodal method of assessment into the study design in order to integrate and compare data from caregiver reports, physical examinations, and the CSA. The CSA enables OPD to be identified through a simple, yet sensitive method, which requires no equipment, avoids exposure to barium and causes little discomfort (i.e., as opposed to videofluoroscopy and videoendoscopy). This instrument for evaluating OPD is being validated in the context of congenital ZIKV infections by another researcher from our group. It should be noted that PAD-PED is a standardized protocol, widely used in Brazilian studies [37–40], which facilitates the comparability of the results. The PAD-PED protocol incorporates information provided by caregivers and from the clinical assessment, while also including an assessment of the muscle tonus, posture and mobility of the stomatognathic system and a functional assessment of swallowing. This approach has broader potential application for the functional assessment of swallowing in the context of ZIKV-exposed pediatric populations. In the context of this study, bias may have been introduced since we cannot guarantee that the clinical evaluator was not influenced by the information provided by the caregivers. However, it is probable that this information had little influence on the conduct of the assessment, as it mainly followed pre-specified and objective criteria established in the protocol. Nonetheless, as the questionnaire was administered to caregivers prior to clinical assessment, caregivers were not influenced by the evaluator's information. A key limitation of this study is that swallowing was assessed for the presence of disorders only in the oral and pharyngeal phases because the esophageal phase could not be clinically assessed using these methods. Although a more invasive method would be necessary for a complete assessment of swallowing, the method used enabled the groups to be compared and was used to guide the conduct of rehabilitation. Thus, in the future, studies using videoendoscopy and videofluoroscopy, with objective examinations of swallowing are recommended in order to provide complementary information on the dysphagic processes in the population affected by ZIKV. Furthermore, longitudinal follow-up of the affected children will be of great value in shedding light on any associations between ZIKV-related microcephaly and malnutrition across the early life course. More broadly, continued follow-up will contribute to understanding of how swallowing difficulties evolve during the course of the child development and inform the accompanying therapeutic strategies and care.

## Conclusion

OPD is a feature of congenital Zika syndrome that may present with varying degrees of severity. These findings indicate that OPD occurs frequently in children with microcephaly, although OPD should also be expected to occur, to a lesser degree, in children with exposure only. Children with OPD, diagnosed through CSA or via caregiver report should be referred for multidisciplinary follow-up and early intervention. Longitudinal follow-up, with an appropriate nutritional assessment and intervention program may help to improve posture and facilitate a safe oral route in mild and moderate OPD.

## Abbreviations

ZIKV: Zika virus
CSA: Clinical swallowing assessment
PAD-PED: Protocol for Assessment of Pediatric Dysphagia
CZS: Congenital Zika syndrome
PCR: Quantitative real-time polymerase chain reaction
WHO: World Health Organization
OPD: Oropharyngeal dysphagia
